# Construction of a new anti-CD19 chimeric antigen receptor and the anti-leukemia function study of the transduced T cells

**DOI:** 10.18632/oncotarget.7079

**Published:** 2016-01-30

**Authors:** Na An, Zhongfei Tao, Saisai Li, Haiyan Xing, Kejing Tang, Zheng Tian, Qing Rao, Min Wang, Jianxiang Wang

**Affiliations:** ^1^ State Key Laboratory of Experimental Hematology, Institute of Hematology and Blood Diseases Hospital, Chinese Academy of Medical Sciences & Peking Union Medical College, Tianjin, China

**Keywords:** chimeric antigen receptor, T cell, CD19, lentivirus, cellular immunotherapy

## Abstract

Chimeric antigen receptor (CAR) transduced T cells have been used to efficiently kill the target tumor cells depending on the single chain variable fragment (scFv) against the specific tumor associated antigen. Here we show the high specific cytotoxicity of the CAR-T cells with very low effector to target cell (E:T) ratio owing to the CD19-scFv, which was constructed in our laboratory and proved to be highly effective in our previous study. Four plasmids containing three generation of CAR were constructed by cloning the CD19-CAR fragment into the lentiviral vector pCDH. CD3 positive T cells were successfully transduced and the CAR protein expression was confirmed by flow cytometry and Western blot. When cocultured with CD19 positive leukemia cell line Nalm-6 cells, CAR-T cells showed specific cytotoxicity: the percentage of target cells decreased to 0 in 24 hours; IL-2, IFN-γ and TNF-α produced in cocultured supernatants increased obviously; and the cytotoxicity reached more than 80%, still remarkable even when the E:T ratio was as low as 1:4. Dynamic change of cell interaction between CAR-T and leukemia cells was visually tracked by using living cells workstation for the first time. A NOD/SCID B-ALL murine model was established using Nalm-6 cells inoculation with a morbidity rate of 100%, and the survival time was prolonged statistically with CAR-T cell treatment. These data demonstrate that the CAR-T cells we prepared could be a promising treatment strategy for CD19 positive tumor diseases.

## INTRODUCTION

Lymphocyte related diseases are very common in hematological malignancies. Acute lymphoblastic leukemia (ALL) occurs mostly in children. Although most of them can obtain complete remission and long term disease free survival through traditional chemotherapy, some still fail to achieve remission and progress to disease relapse [1-3]. The overall survival for adults with ALL is quite poor [4, 5]. Allogeneic hematological stem cell transplantation (HSCT) is considered to be a curative therapy, but the rare matched donor and severe graft versus host disease (GVHD) hinder the general use of it.

Cellular immunotherapy is a new treatment strategy. The chimeric antigen receptor transduced T (CAR-T) cells have been considered to be a promising therapy recently [6-8]. CAR is a fusion protein connected by the following parts sequentially. The scFv is the light chain connected directly with the heavy chain of an antigen specific antibody responsible for recognizing the tumor specific antigen. The costimulatory molecules including CD28, OX40, CD137 and so on are important for T cell activation and proliferation. The CD3ζ chain is the last part of signal transduction region to induce the final activation of CAR-T cells. The CAR is artificially classified into three generations depending on the number of costimulatory molecules it consists. The second generation consisting of only one or the third generation of two or more costimulatory molecules are used most widely. Once the scFv recognizes the specific antigen, CAR-T cell is activated and lyses the tumor cells. This is not MHC restricted and is highly antigen specific [9-11], with no risk of GVHD development as usually occurred in HSCT.

CD19 is expressed on the surface of almost all B cell malignancies, but not on the hematopoietic stem cells and other tissue cells [12], so it has been an ideal target. Many centers have designed their own CD19-CAR-T cells proved to be effective and safe after testing in clinical trials [13-23].

Since the efficiency of CAR-T cells may be different due to the specific antigen recognition epitopes of scFv, and we had constructed a highly effective CD19 specific scFv (CD19-scFv) in our previous study, which was used to construct our own CAR plasmids in this study. By preparing high quality lentivirus, CD3 positive T cells were successfully transduced and the function was confirmed using the CD28-CD3ζ-CAR-T cells as an example. In *in vitro* function study, we discovered that a very small amount of CAR-T cells were needed to lyse large number of target cells, which was different from most other reports requiring high E:T ratio. And we used living cells workstation for the first time to visually track cell interaction between CAR-T and leukemia cells. The xenograft mice model also showed anti-leukemic effect and safety *in vivo*.

## RESULTS

### Four CD19-CAR lentiviral vectors were constructed

**Figure 1:**
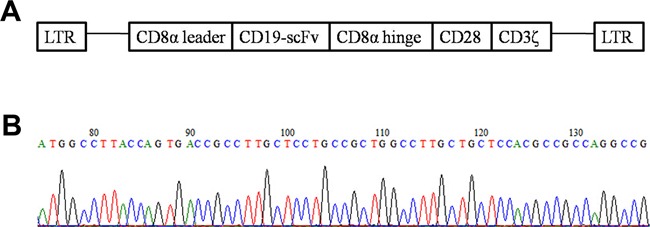
Structure diagram of CD19-CAR plasmid and part of the sequencing results **A.** Structure diagram of the lentivial vector CD19scFv-CD828ζ. (LTR, long terminal repeat; CD8α leader, sigal peptide of CD8α; scFv, single chain variable fragment; CD8α hinge, hinge region of CD8α; CD28, transmembrane and cytoplasmic domain of CD28; CD3ζ, cytoplasmic region of CD3ζ). **B.** Part of the sequencing results, the first 21 amino acids of CD8α leader.

We successfully constructed four kinds of CD19-CAR lentiviral vectors using the CD19-scFv previously constructed in our laboratory. The one contained CD28 costimulatory domain named CD19scFv-CD828ζ was used for the following function study. The sequences were combined in order of CD8α leader, CD19-scFv, CD8α hinge region, CD28 transmembrane and cytoplasmic domain and the CD3ζ cytoplasmic region (Figure 1A). Part of the sequencing results were shown in Figure 1B.

### T cells were successfully transduced and expressed CAR protein

**Figure 2:**
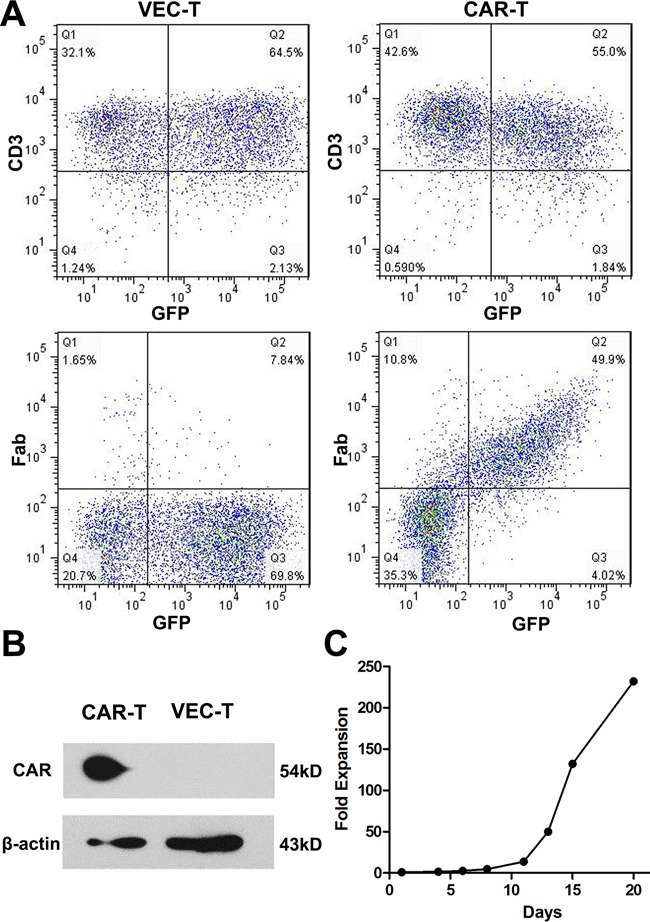
CAR protein expressed on transduced T cells and the CAR-T cells proliferated efficiently **A.** Flow cytometry to detect the CD3 and Fab expression on cell surface 4 days after virus transduction. CAR transduced cells (CAR-T, right panel) were more than 95% of CD3^+^ (right upper panel) and 50% of GFP^+^Fab^+^ (right lower panel), while empty vector transduced cells (VEC-T, left panel) were GFP^+^Fab^−^. **B.** Western blot analysis of cell lysates from CAR-T and VEC-T cells using an antibody specific against CD3ζ (CAR protein is 54kD, while natural CD3ζ is 16kD). β-actin was used as a loading control. **C.** CAR-T cells were counted by trypan blue staining every 2∼3 days after transduction. Fold expansion at each time point was calculated by dividing the cell number on transduction day.

CAR-T cells could express both GFP and CAR protein while empty vector transduced T (VEC-T) cells express GFP only. As shown in Figure 2A, cells were more than 95% of CD3^+^ after transduction and the GFP^+^Fab^+^ CAR-T cells were detected through flow cytometry. The transduction efficiency was about 50%. So the GFP^+^ cells could represent the CAR^+^ cells. To further confirm the expression of CAR protein on GFP^+^ cells, we sorted the GFP^+^ T cells and performed the Western blot, the GFP^+^ T cells of VEC-T cells were used as control. A 54kD fusion protein was detected in CAR-T cells (Figure 2B). We also study the proliferation efficiency of CAR-T cells by counting the number changes of CAR-T cells using trypan blue staining. The cells could proliferate more than 100 folds in 2 weeks under our culture system (Figure 2C).

### CAR-T cells showed specific cytotoxicity against CD19^+^ leukemia cells

**Figure 3:**
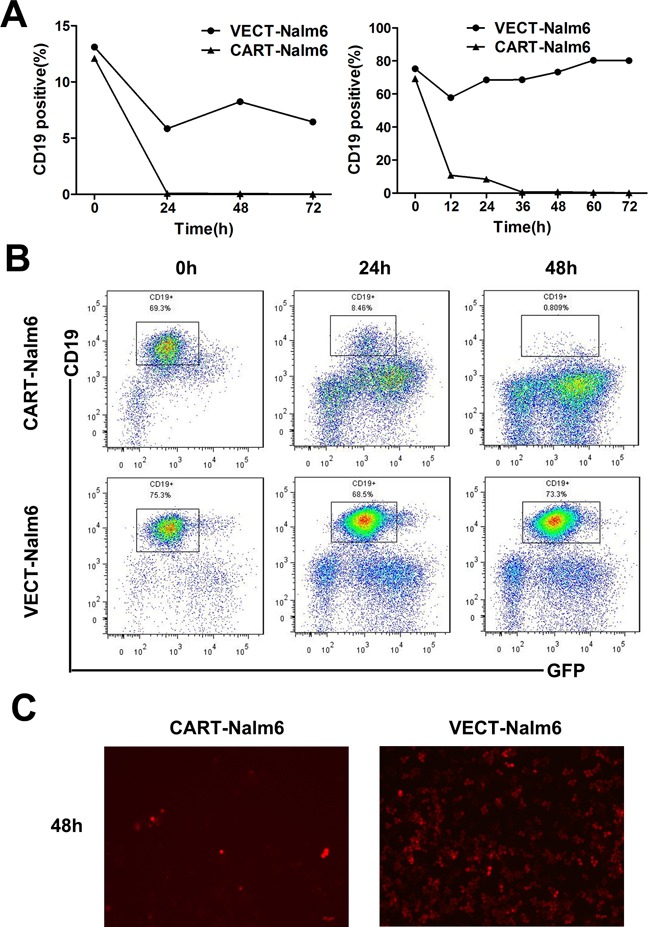
Nalm-6 leukemia cells could be eliminated by CAR-T cells **A.** Percentage of CD19^+^ Nalm-6 cells detected by flow cytometry at different time points when cocultured with CAR-T cells or VEC-T cells as control. E:T ratio was 6:1 (left panel) or 1:3 (right panel). **B.** Diagram of flow cytometry showing the residual Nalm-6 cells when cocultured with CAR-T cells (upper panel) or VEC-T cells (lower panel) for 0h, 24h and 48h. **C.** Fluorescence microscopy of RFP^+^ Nalm-6 cells after cocultured with CAR-T cells (left panel) or VEC-T cells (right panel) for 48 hours (magnification, 10×).

**Figure 4:**
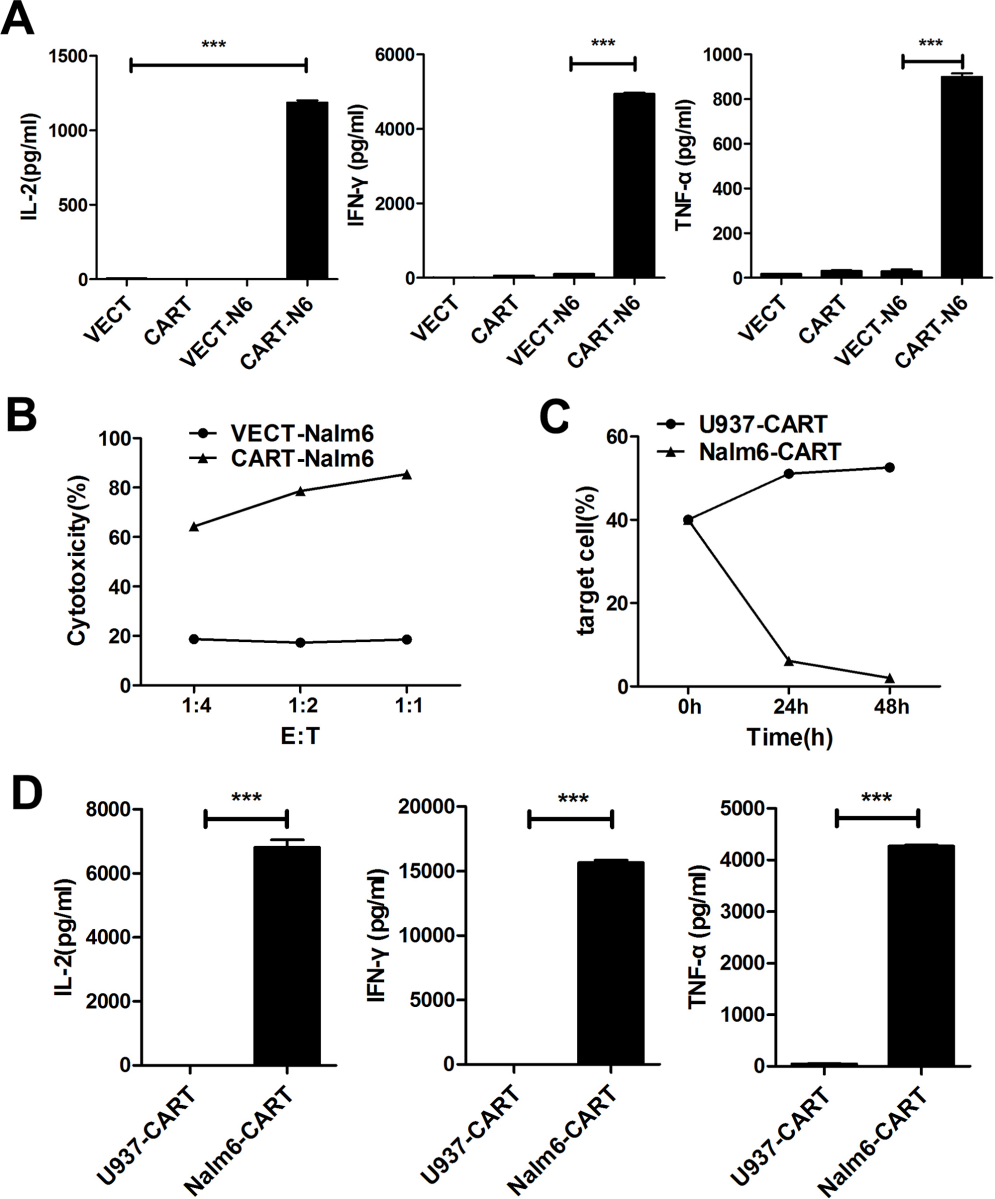
CAR-T cells showed specific cytotoxicity against CD19^+^ Nalm-6 cells but no effect on CD19^−^ U937 cells **A.** ELISA detection of IL-2, IFN-γ and TNF-α in the cultured supernatants of VECT (VEC-T), CART (CAR-T), VECT-N6 (VEC-T cocultured with Nalm-6) and CART-N6 (CAR-T cocultured with Nalm-6) cultured for 24 hours. Results are shown as mean±SD from three independent experiments. **B.** CytoTox96^®^ Non-Radioactive Cytotoxicity Assay of the cell cytotoxicity at different E:T ratios. The cytolytic activity was measured by assays of lactate dehydrogenase (LDH) release after cocultured for 7h using the detection kit according to the manufacturer's instructions. Cytotoxicity is reported as the mean±SD of triplicate determinations. **C.** Flow cytometry detection of the percentage of target cells (U937 or Nalm-6) cocultured with CAR-T cells at different time points. **D.** ELISA detection of different cytokines in U937-CART (U937 and CAR-T) or Nalm6-CART (Nalm-6 and CAR-T) coculture system after 24 hours. Results are shown as mean±SD from three independent experiments.

In *in vitro* assay of the specific cytotoxicity of CD19-CAR-T cells, we used CD19^+^ Nalm-6 leukemia cells as target cells and CD19^−^ U937 leukemia cells as control target cells. Compared to VEC-T cells, CAR-T cells showed obvious cytotoxicity against Nalm-6 cells. As showed in Figure 3A, no matter the E:T ratio was as high as 6:1 or as low as 1:3, the CD19^+^ cells could not be detected by flow cytometry after 24 hours of coculture, but persisted in the control group even after 72 hours. And the flow charts were shown (Figure 3B). The difference of cells density was also observed under fluorescence microscope after 48 hours (Figure 3C), in which the red-colored cells represented residual Nalm-6 cells transfected with red fluorescent protein (RFP). Since the increase of cytokines concentration is the response of T cells activation and cytotoxicity, we detected the classic cytokines of IL-2, IFN-γ and TNF-α as an example to evaluate the activation efficacy of CAR-T cells cocultured with target cells. The concentrations of IL-2, IFN-γ and TNF-α were (1186.34±15.5)pg/ml, (4943.93±29.46)pg/ml and (899.345±15.72)pg/ml in the supernatant of Nalm6-CART coculture system, respectively, all were significantly higher than that of control groups (*P*<0.001) (Figure 4A). CytoTox96^®^ Non-Radioactive Cytotoxicity Assay based on the LDH release showed that even a small amount of CAR-T cells could kill a large number of Nalm-6 cells. When E:T ratio was as low as 1:4, the cytotoxicity could be more than 60% and increased as the E:T ratio increase (Figure 4B). The above effects were not observed in targeting U937 cells. As showed in Figure 4C, the proportion of the Nalm-6 cells dropped dramatically in the coculture system, while that of the U937 cells kept raising in the coculture system over time. And the cytokines could not be detected in U937-CART cells, which was significantly different from that of Nalm6-CART cells (Figure 4D).

### Target cells lysis were observed by microscopic imaging

**Figure 5:**
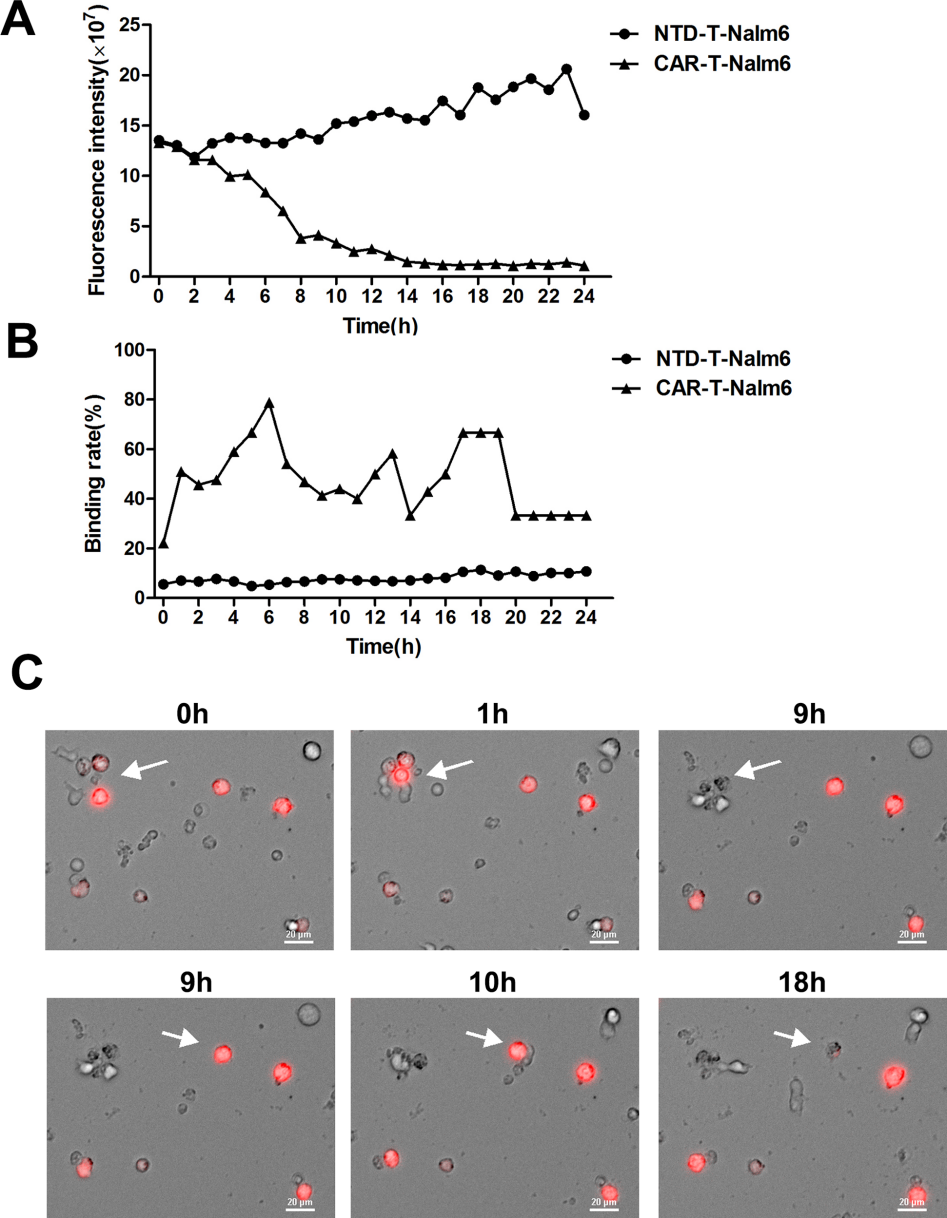
Dynamic observation of cell interaction by using living cells workstation **A.** Fluorescence intensity of RFP^+^ Nalm-6 cells changes over time analyzed by Elements 3.1 and Velocity 6.1 software. CAR-T-Nalm6 represents Nalm-6 cells cocultured with CAR-T cells, while NTD-T-Nalm6 represents Nalm-6 cells cocultured with none transduced T cells (NTD-T). **B.** Binding rates of Nalm-6 cells to CAR-T cells or NTD-T cells at different time points were analyzed by Elements 3.1 and Velocity 6.1 software. **C.** Photographs representing the process of cell binding and lysis (arrows) captured by living cells workstation (Nikon Ti-e). Red-colored cells were RFP^+^ Nalm-6 cells and others were CAR-T cells.

Nalm-6 cells transfected with RFP were cocultured with CAR-T cells or none transduced T (NTD-T) cells and immediately exposed to the microscopic imaging system. Videos tracking the cell interaction over time were made. Gradually disappeared red-colored cells representing Nalm-6 cells lysis were observed in the CAR-T group (Supplementary Video S1), but not in the NTD-T group (Supplementary Video S2). Red fluorescence intensity in CAR-T group decreased sharply in 8 hours but kept increasing in control group (Figure 5A). As shown in Figure 5B, dynamic binding rates in CAR-T group were remarkably higher than that of control group. Photographs of typical cell interaction including binding and then lysis were shown in Figure 5C and the corresponding video was shown in Supplementary Video S3. All of the above visually tracked study showed the direct binding and killing of target cells by CAR-T cells.

### CAR-T cells prolonged survival of B-ALL mice

**Figure 6:**
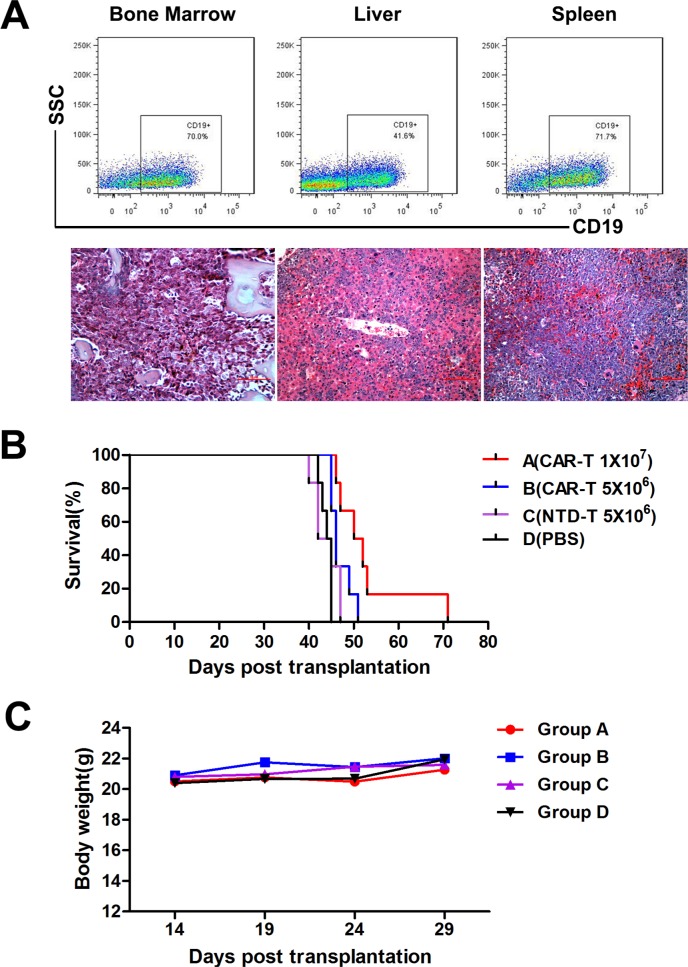
*In vivo* CAR-T cell treatment in murine B-ALL model **A.** Detection of human CD19^+^ cells by flow cytometry (upper panel) and histopathologic analysis (lower panel) of bone marrow, liver and spleen from Nalm-6 cells inoculated mice. **B.** Kaplan-Meier survival curves of four treatment groups. The *P*-values were determined by log-rank test. *P*<0.05 when group A compared with all other groups. *P*=0.009 when group B compared with group D, but no difference between group C and D (*P*=0.611). **C.** Average body weight of four groups (n=6) after CAR-T cell treatment. CAR-T cells were administered at day 15 after Nalm-6 cells inoculation.

To evaluate the *in vivo* function of CAR-T cells, we established a B-ALL mouse model using Nalm-6 cells inoculation. All transplanted mice developed aggressive acute lymphocytic leukemia with extensive infiltrations of CD19^+^ human cells in hematopoietic organs confirmed by flow cytometry and pathology (Figure 6A). The mean survival times of CAR-T cell treatment groups were prolonged significantly compared to that of control groups (Figure 6B). Mean survival times of Group A, B, C and D were (53.167±3.736) d, (47.000±1.000) d, (43.833±1.195) d and (44.000±0.516) d, respectively. CAR-T treated Group A mice showed a longer survival time compared to all other groups (*P*<0.05). Survival time of lower dose CAR-T treated Group B mice was also prolonged compared to that of Group D (*P*=0.009), but NTD-T cells could not prolong the survival time of Group C mice compared to that of Group D (*P*=0.611). Thus demonstrated the anti-leukemia function of CAR-T cells *in vivo* and the efficiency could be improved when enough cells were used. No rapid body weight decrease (Figure 6C) or other adverse effect were observed in all groups, indicating the safety of CAR-T cell treatment.

## DISCUSSION

The cellular immune therapy has become a promising strategy in treatment of B cell malignancies. And the newly reported CAR-T cells have been proved to be incredible effective. The second generation CAR-T cells containing CD28 or CD137 costimulatory molecules are commonly used at present. Represented by the National Cancer Institute (NCI), Memorial Sloan-Kettering Cancer Center (MSKCC) and so on, the clinical application of CD28-CAR-T cells is practicable. Although University of Pennsylvania (Upenn) center used the costimulatory molecule of CD137 in their CD137-CAR-T cells instead of CD28, there's no definite conclusion about which structure is better [24]. When CAR-T cells start to work, the first step is the specific recognition and binding of scFv to tumor antigen, which leads to the following CAR modified T cell activation and cytotoxicity, so it's rational to consider that the difference in antigen recognition epitopes of scFv can also affect the function of CAR-T cells. The effect of CD19-scFv antibody against B lymphocyte malignancy has been confirmed in our previous study, we tried to investigate its function as a constituent part of the new CAR in the CAR-T cell application. Four CD19-CAR lentiviral vectors were successfully constructed. Here we focused only on CD28-CAR-T cell function *in vivo* and *in vitro*, providing the basis for the following clinical application.

The function of CAR-T cells was comprehensively tested from three aspects, including the residual leukemia cells monitoring by flow cytometry, cytokines levels detecting by ELISA and cytotoxicity assay by LDH release. All results consistently showed strong cytotoxicity of the CD19-CAR-T cells against CD19^+^ leukemia cells. When E:T ratio was only 1:3, targeted leukemia cells could be cleared within 24 hours. Cytotoxicity could reach above 60% with an E:T ratio as low as 1:4. This was different from most other reports requiring more CAR-T cells, i.e. the higher E:T ratio. CD19^−^ U937 leukemia cells were used as control target cells to confirm the specificity.

We visually track the dynamic change of CAR-T cells interacting with target cells, and the process of cell binding and lysis was clearly observed by using the living cells workstation for the first time. To further evaluate the effect of CAR-T cells *in vivo*, we successfully established a B-ALL murine model with a morbidity of 100%. CAR-T cell treatment extended the survival of mice compared with that of control groups, the efficacy was more obvious with a higher dosage of CAR-T cells. The mice did not achieve long term disease-free survival, possibly due to the failure of CD28-CAR-T cells to proliferate persistently *in vivo*. But the effect could be improved by increasing cell numbers and infusion frequencies of CAR-T cells, and at the same time to avoid the risk of persistent B cells aplasia. Function of CAR-T cells are influenced by many factors, including the CAR structure (scFv and costimulatory molecules, etc), culture condition *in vitro* and T cell subtype [25-29], infusion time, dose and frequency, and usage of other drugs (chemotherapy, IL-2 and PD-1 inhibitors, etc). Therefore, we will optimize various conditions to gain the best *in vivo* treatment effect in future.

In conclusion, we successfully constructed four new CD19-CAR lentiviral vectors and transduced T cells. The CAR-T cells showed strong specific cytotoxicity against CD19^+^ leukemia cells in comprehensive function study *in vitro*. Preliminary animal experiments also indicated its anti-leukemia effect *in vivo*, and further study is needed to optimize the therapeutic efficacy.

## MATERIALS AND METHODS

### Construction of anti-CD19 CAR lentiviral vectors

The mouse anti-human CD19-scFv segment was cloned from the PET22b plasmid containing the V_L_-linker-V_H_ sequence generated by our laboratory before. The scFv sequence optimized suitable for expression in human cells was linked to CD8α signal sequence with NheI and EcoRI sites at both ends. The sequences of CD8α hinge and transmembrane region, the CD28 transmembrane and cytoplasmic domain and the CD137 or CD3ζ cytoplasmic region were all referred to the NCBI GeneBank based on other reports. The above signal transduction regions were all amplified from the cDNA of a healthy donor's peripheral blood mononuclear cells (PBMC) by PCR and connected in order by overlap PCR. And EcoRI and NotI sites were included. The two parts were finally connected by enzyme digestion and ligation, and the entire sequence was cloned into the lentiviral vector pCDH (System Bioscience, USA) with GFP marker gene, named pCDH-CAR plasmid. All sequences were confirmed by gene sequencing (Life Technologies, China). The empty vector pCDH was used as control vector.

### Lentivirus production

To produce lentiviruses, 293T packaging cells were transfected with empty vector or pCDH-CAR plasmids as mentioned above. The packaging plasmids were psPAX2 and pMD2.G (Invitrogen, USA). The day before transfection, 293T cells were plated in the 10cm dish cultured in DMEM (Invitrogen, USA) with 10% FBS (Hyclone, USA). When cell density reached 60%∼80%, the cells were transfected with the above plasmids using polyethylenimine (PEI, Polysciences, USA). After the transfected cells incubated in 37°C, 5% CO_2_ incubator for 12∼16 hours, the culture medium was replaced with 5∼6ml fresh DMEM with 10% FBS. The supernatants containing viruses were harvested at 24 hours and 48 hours and concentrated by ultracentrifugation for 90 minutes at 50,000g, 4°C, then stored at −80°C until use.

### T cells isolation and transduction

Peripheral blood of healthy donors was obtained from Tianjin Blood Center. CD3 positive T cells were isolated and enriched by CD3 enrichment antibody cocktail (Stem Cell, USA) and Ficoll solution (TBD Science, China). The cell purity was detected by flow cytometry (CantoII, BD, USA) stained with APC-labeled mouse anti-human CD3 antibody (Biolegend, USA). T cells maintained in RPMI 1640 (Invitrogen, USA) plus 10% FBS were inoculated in 24-well plates embedded with CD3 and CD28 antibody (eBioscience, USA) with a cell density of 1∼1.5×10^6^/ml, and rhIL-2 (R&D, USA) was added with a dose of 200U/ml. After 24 hours, cells were transduced with thawed lentiviruses which were added directly to the plate. 8μg/ml polybrene (Sigma, USA) was added. The plate was then centrifuged for 90 minutes at 1800rpm, 32°C and incubated for another 24 hours without disturbing. The other day, the transduction was repeated once. Then culture medium was changed every 2 days and cells were kept in flasks with a density of 1.0×10^6^/ml with rhIL-2 routinely used.

### CAR protein detection on transduced cells

Flow cytometry and Western blot analysis were done to confirm the expression of CAR protein. In flow cytometry, transduced cells were harvested and washed once with PBS, stained with biotin-labeled goat anti-mouse IgG, F(ab')_2_ antibodies (Jackson Immunoresearch, USA) for 20 minutes at 4°C and washed twice. Then APC/Cy7-conjugated streptavidin antibodies (Biolegend, USA) were added, incubated at 4°C for another 20 minutes and washed once. Then the cells were analyzed by flow cytometry and results were analyzed with FlowJo software. In Western blot analysis, GFP positive cells were sorted by BD FACS AriaIII System and lysed with buffer containing 50 mMTris/HCl (pH 7.5), 150 mMNaCl, 1% Triton X-100, 1% sodium deoxycholate, 0.1% SDS and 0.1mM PMSF. Proteins were analyzed by SDS-PAGE and Western blotting. Membranes were incubated with primary antibodies against human CD3ζ (BD Pharmingen, USA) and β-actin (Cell Signaling Technology, USA). Blots were visualized using the ECL (Millipore, USA) with Image Quant LAS-4010 (GE Healthcare, USA). The empty vector transduced cells were used as control.

### *In vitro* function study of CAR-T cells

In order to verify the specific cytotoxicity of CAR-T cells against CD19^+^ leukemia cells, a B-cell acute lymphoblastic leukemia cell line Nalm-6 cells were used as the target cell. VEC-T cells were used as control effector cells. Effector and target cells were cocultured in 24-well plates with an E:T ratio of 6:1 or 1:3. Repetitive wells were set for detection at different time points (0h, 12h, 24h, 36h, 48h, 64h and 72h). Each time point, cells were harvested and washed once, stained with PE/Cy7-conjugated mouse anti-human CD19 antibody (Biolegend, USA) for 20 minutes on ice, then washed and resuspended in PBS for flow cytometry analysis. The percentage of CD19^+^ cells represented the residual level of target cells. The supernatants were harvested and stored as aliquots at −20°C for the assay of cytokines produced in the coculture system, including IL-2, IFN-γ and TNF-α using ELISA kits (R&D, USA). The supernatants from CAR-T cells or VEC-T cells cultured alone were also harvested and used as control groups to exclude the natural cytokine release. Absorbance was measured with Synergy H4 Hybrid Microplate Reader (Biotek, USA) at 570 nm. CytoTox96^®^ Non-Radioactive Cytotoxicity Assay (Promega, USA) was further used to assess the specific lysis of target cells according to the manufacture's protocol with the E:T ratio of 1:4, 1:2 and 1:1. Three repeated wells were designed. After 7 hours, the OD value was detected at 490 nm and cytotoxicity was calculated according to the formula. In order to verify the specific cytotoxicity of CAR-T cells, acute myelomonocytic leukemia cell line U937 cells were used as control target cells in above experiments.

### Dynamic imaging of cell interaction by living cells workstation

To monitor the dynamic change of cells in the coculture system, RFP transfected Nalm-6 cells were mixed with CAR-T cells or NTD-T cells at 1:1, respectively. A total volume of 400μl cell mixture was inoculated in a 24-well plate for 1 minute at room temperature. Then bright field and TRITC red channel video was captured by the living cells workstation (Nikon Ti-e, Japan) for 24 hours. The exposure time was 5s and pictures were taken every 10 minutes under 37°C, 5%CO_2_. RFP fluorescence intensity and cell binding rate changes over time were analyzed by Elements 3.1 and Velocity 6.1 software. The percentage of Nalm-6 cells bound to T cells in all Nalm-6 cells in one field was defined as binding rate.

### Establishment of murine B-ALL model and *in vivo* treatment with CAR-T cells

To establish the B-ALL mouse model, 4 weeks old NOD/SCID female mice were purchased from Institute of Laboratory Animal Sciences (CAMS&PUMC, China). After adaptive feeding for 2 weeks, 24 mice were irradiated at 200 cGy and intravenously inoculated with 5×10^5^ Nalm-6 cells. Two weeks after transplantation, 1% CD19^+^ human cells were detected in peripheral blood by flow cytometry. Then the mice were randomized into four treatment groups. Group A were injected intravenously with 1×10^7^ CAR-T cells, Group B with 5×10^6^ CAR-T cells, Group C with 5×10^6^ NTD-T cells and Group D with the equivalent volume of PBS. The disease development was assessed by percentages of circulating CD19^+^ leukemia cells by flow cytometry. Symptoms including diarrhea, paralysis and body weight loss were also monitored. The overall survival was measured from the date of transplantation until death. Dead mice were dissected for pathological and flow cytometry analysis to confirm the diagnosis of leukemia. All animal experiments were approved by the Institutional Animal Care and Use Committee of Peking Union Medical College.

### Statistical analysis

SPSS software (version 16.0) was used for statistical analysis. The comparisons were performed by Student's *t*-test analysis using GraphPad Prism (version 5.0). The lifespan of mice was analyzed by Kaplan-Meier methods and a log-rank test. *P*-values<0.05 were considered statistically significant. Briefly, *P< 0.05, **P< 0.01, and ***P< 0.001 in comparison.

## SUPPLEMENTARY VIDEOS


