# Supplementary material for: Construction of a new anti-CD19 chimeric antigen receptor and the anti-leukemia function study of the transduced T cells
Source: Oncotarget. 2016 Jan 30;7(9):10638–49. doi: 10.18632/oncotarget.7079 (PMC4891147; doi:10.18632/oncotarget.7079)
Supplement: Supplementary file 1 [file oncotarget-07-10638-s001.pdf]

SUPPLEMENTARY VIDEOS

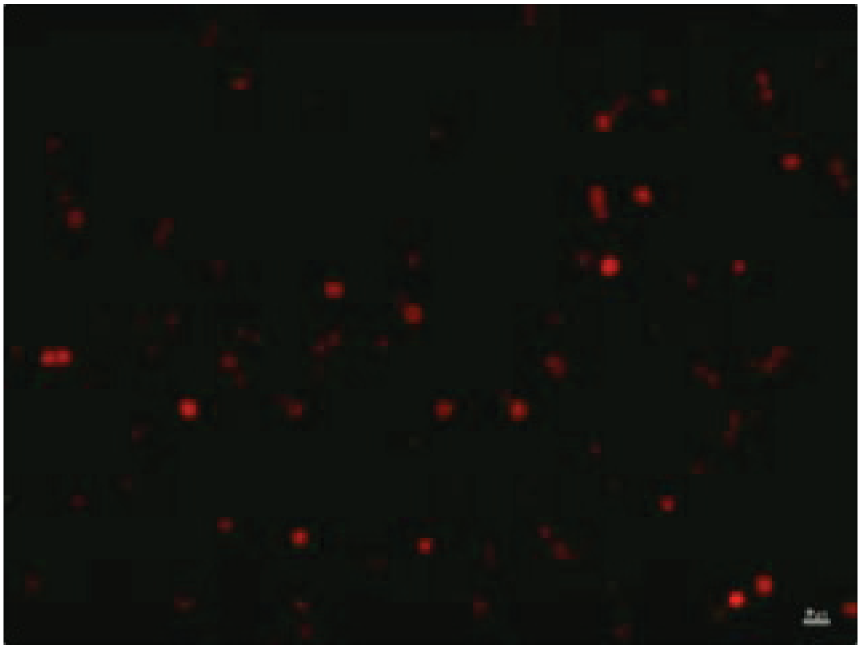

Supplementary Video S1: RFP$^+$ Nalm-6 cells coculturing with CAR-T cells were real-time monitored by living cells workstation for 24 hous.

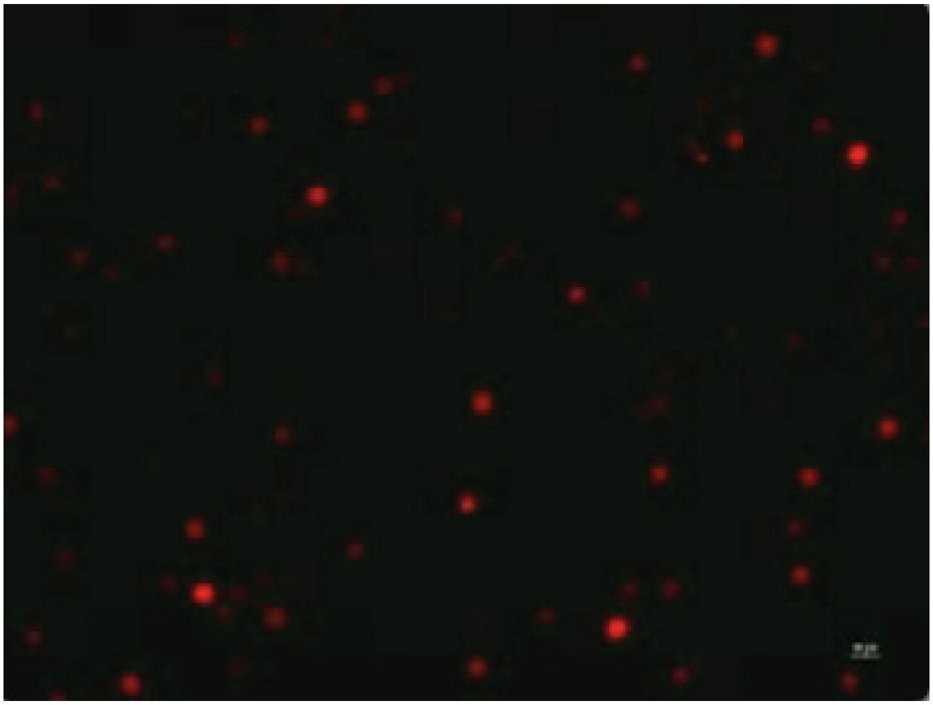

Supplementary Video S2: RFP$^+$ Nalm-6 cells coculturing with NTD-T cells were real-time monitored by living cells workstation for 24 hous.

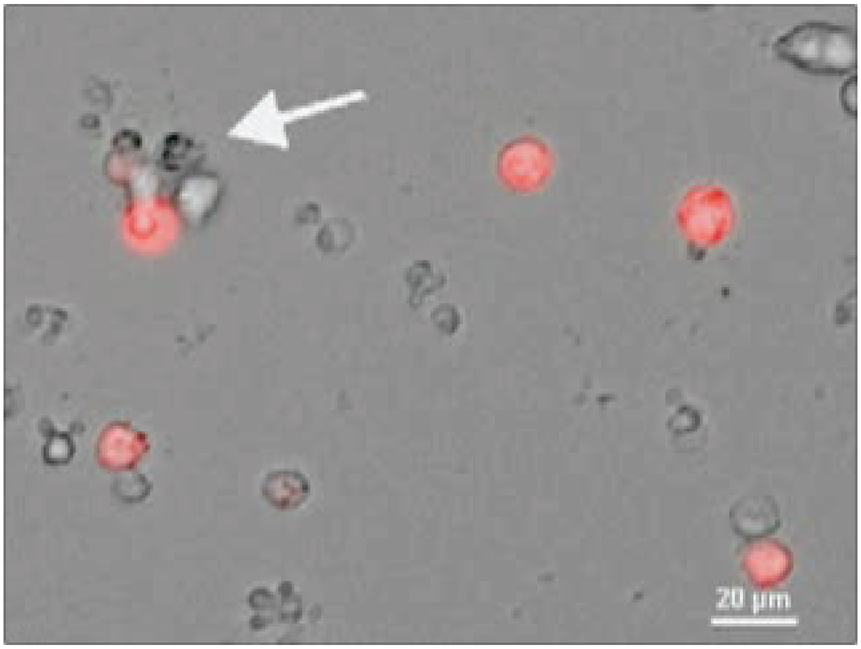

Supplementary Video S3: Process of RFP⁺ Nalm-6 cells interacted and lysed by CAR-T cells were captured by living cells workstation.
